# Mouse Model Resources for Vision Research

**DOI:** 10.1155/2011/391384

**Published:** 2010-10-31

**Authors:** Jungyeon Won, Lan Ying Shi, Wanda Hicks, Jieping Wang, Ronald Hurd, Jürgen K. Naggert, Bo Chang, Patsy M. Nishina

**Affiliations:** The Jackson Laboratory, 600 Main Street, Bar Harbor, ME 04609, USA

## Abstract

The need for mouse models, with their well-developed genetics and similarity to human physiology and anatomy, is clear and their central role in furthering our understanding of human disease is readily apparent in the literature. Mice carrying mutations that alter developmental pathways or cellular function provide model systems for analyzing defects in comparable human disorders and for testing therapeutic strategies. Mutant mice also provide reproducible, experimental systems for elucidating pathways of normal development and function. Two programs, the Eye Mutant Resource and the Translational Vision Research Models, focused on providing such models to the vision research community are described herein. Over 100 mutant lines from the Eye Mutant Resource and 60 mutant lines from the Translational Vision Research Models have been developed. The ocular diseases of the mutant lines include a wide range of phenotypes, including cataracts, retinal dysplasia and degeneration, and abnormal blood vessel formation. The mutations in disease genes have been mapped and in some cases identified by direct sequencing. Here, we report 3 novel alleles of *C* *r* *x* ^*tvrm*65^, *R* *p*1^*tvrm*64^, and *R* *p* *e*65^*tvrm*148^ as successful examples of the TVRM program, that closely resemble previously reported knockout models.

## 1. Introduction

The Eye Mutant Resource (EMR) and the Translational Vision Research Models (TVRMs) programs currently housed at The Jackson Laboratory are tailored to provide genetically defined models of vision-associated diseases to the Research Community. The EMR screens for spontaneous mutations in the large production and repository colonies, while the TVRM program screens for chemically induced mutations in third-generation (G3) offspring of mutagenized mice. Both programs are motivated by the need for well-characterized models for studying the function of particular molecules in the eye, for examining disease pathology, and for providing a resource to test therapeutic regimens. 

In the early phases of the EMR program, the tools for examining mice for ocular abnormalities were adapted for the small size of the mouse eye [1, 2]. These tools included indirect ophthalmoscopy, slit lamp biomicroscopy, fundus photography, and electroretinography (ERG). Initially, mice from various stocks and inbred strains were screened to identify spontaneous ocular mutants using the first two methodologies. Currently, ERG screening is done as well to identify and characterize new retinal mutants. As secondary screens, fluorescein angiography is used to detect vascular changes [2], and noninvasive tonometry [3] is used to assess changes in intraocular pressure. Screening has also been expanded to include genetically engineered strains from the Jackson Laboratory's Genetic Resource Sciences (GRS) repository that are systematically examined as they are removed from the shelf or are retired from breeding. Also, in addition to the initial phenotypic characterization, the EMR strives to identify the mutations underlying the disorders. 

Systematic chemical mutagenesis screens have been successfully carried out in several model organisms, including *Drosophila* [4], *C. elegans* [5], and zebrafish [6, 7]. The zebrafish screens have provided valuable eye models, especially those pertaining to eye development [8]. In addition to our efforts, other mutagenesis screens for eye phenotypes in mice have been reported in which a number of mutants have been described [9–11]. Although different methods for mutagenizing mice are available, the alkylating agent, N-ethyl-N-nitrosourea (ENU), is the mutagen most commonly used [12]. ENU mainly induces point mutations resulting in a range of consequences including total or partial loss-of-function, dominant-negative, or gain-of-function alleles [13–16]. Its effectiveness as a mutagen is dependent on dosage, frequency of administration, and mouse strain. Effectiveness, in terms of identifying mutants, depends upon the type of screen (e.g., dominant versus recessive) and the reproducibility of the phenotypic assay utilized. Mutant recovery has ranged from a rate of 1/175 [17], to ~1/666 in Specific Locus Tests (SLTs) [12], and to an average of 1/1470 based on recessive screening in a defined chromosomal region [18]. The mutation rates for individual loci can vary by almost tenfold [12, 17, 18]. 

The majority of large-scale mutagenesis screens have been dominant screens. This is probably due to the relative ease of creating mutagenized mice for dominant screens compared to recessive ones. Screening for dominants on a genome-wide basis can be done in one generation (G_1_), while recessives generally require three. The Neuherburg Cataract Mutant Collection of ~170 dominant mutants was assembled through screening over 500,000 first-generation mice exposed to various mutagens [19]. The GSF-Munich [14] and MRC-Harwell [13, 20] programs were established using a phenotype-based approach to screen thousands of mice for dominant mutations affecting a variety of biological processes. A major drawback to dominant screens, however, is that not all mutations have dominant effects. A dominant screen will, therefore, miss many of the induced mutations. Estimates suggest that the frequency of diseases caused by recessive mutations is 4–10-fold higher than for dominant ones. In fact, of 218 eye mutants surveyed in the Mouse Genome Informatics Database, 80% were recessive mutations and only 20% were dominant or semidominant. Therefore, the TVRM program screened a G3 population of mutagenized mice for recessive mutations.

Screening for spontaneous and chemically induced mutants provides an important source of models to study the effects of single-gene mutations found in human patients. Additionally, new mutations within the same gene provide allelic series in which splice variants or domain-specific effects can be queried. Finally, mutations in novel genes that lead to retinal disorders can be discovered using a forward genetic approach.

## 2. Materials and Methods

### 2.1. Origins of Mice and Husbandry

**Figure 1:**
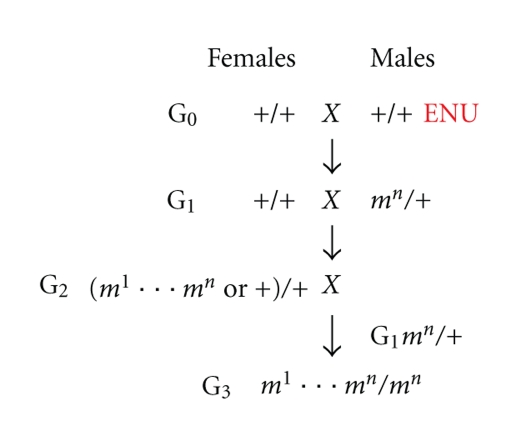
Schematic representation of the mating scheme of dominant (G_1_) or recessive (G_3_) screens. Male mice were mutagenized (3 weekly doses, 80 mg/kg) and mated to WT females after 4 weeks. If any female was pregnant within 5 weeks, the mating was discarded. If, however, male mice impregnated a female after that, the resulting G_1_ males were crossed to their respective female counterparts, and the G_2_ progeny were backcrossed to the G_1_ fathers to generate G_3_ offspring.

The ages at which the visual system is affected by disease can vary considerably. For the EMR program, an initial screen of JAX Mice & Services (JMSs) production colonies and mice removed from the GRS Repository is routinely performed at ~2 months of age and if necessary, additional screening is done at an older age, usually at 6 months of age. Also, as with other neuronal diseases, diseases of the visual system are not reversible, so ocular diseases can be captured in retired breeders. Therefore, when available, retired breeders that are older than 1 year of age are screened. C57BL/6J (B6) G3 ENU mutagenized mice were screened by the TVRM program. For the chemically induced mutations, ENU was administered to male B6 mice in three weekly injections of 80 mg/kg. G3 offspring were generated using a three-generation backcross mating scheme (Figure 1). G3 mice were screened at 24 weeks of age in order to enhance our ability to identify late onset diseases.

To determine if the disease phenotype was inheritable, mutant mice were outcrossed to wild-type (WT) mice to generate F1 progeny with subsequent intercrossing of the resultant F1 mice to generate F2 progeny. Both F1 and F2 mice were examined by indirect ophthalmoscopy or ERG. If F1 mice were affected, the pedigree was designated as segregating a dominant mutation. If F1 mice were not affected but ~25% of F2 mice were affected, the pedigree was designated as segregating a recessive mutation. Once the observed ocular phenotype was determined to be genetically heritable, mutants were bred and maintained in the Research Animal Facility at JAX. Mice were provided with NIH 6% fat chow diet and acidified water, with 12:12 hour dark:light cycle in pressurized individual ventilation caging which are monitored regularly to maintain a pathogen-free environment. Procedures utilizing mice were approved by the Institutional Animal Care and Use Committee.

### 2.2. Clinical Evaluation and Electroretinography

Mice, dark adapted for a minimum of 1 hour, were treated with atropine prior to examination by indirect ophthalmoscopy with a 60 or 78 diopter aspheric lens. Fundus photographs were taken with a Kowa small animal fundus camera using a Volk superfield lens held 2 inches from the eye as previously described [2]. 

For electroretinographic evaluation of mutants, following a 2-hour dark adaptation, mice were anesthetized with an intraperitoneal injection of xylazine (80 mg/kg) and ketamine (16 mg/kg) in normal saline. Additional anesthetic was given if akinesia was inadequate. The equipment and protocol used here were those previously described [21]. Briefly, dark-adapted, rod-mediated ERGs were recorded with the responses to short-wavelength flashes over 4.0-log unit to the maximum intensity by the photopic stimulator. Cone-mediated ERGs were recorded with white flashes after 10 min of complete light adaptation. The signals were sampled at 0.8 msec intervals and averaged.

### 2.3. Genetic Mapping

Genomic DNA was isolated from tail tips using a PBND (PCR buffer with nonionic detergents) preparation, which was adapted from a protocol from Perkin Elmer Cetus [22]. Tail tips were digested in PBND buffer + Proteinase K overnight at 55°C. Samples were heated to 95°C for 10 minutes, and 1 *μ*L of the DNA preparation was used in a 12 *μ*L PCR reaction. Amplicons were visualized with ethidium bromide after electrophoretic separation on a 4% agarose gel.

For mapping purposes, phenotypically affected mice, presumed to be homozygous for the mutations, were mated with DBA/2J mice. The resulting F1 offspring were intercrossed to generate F2 offspring if recessive and backcrossed (BC) to WT parental if dominant. Resulting progeny were phenotyped by indirect ophthalmoscopy. DNA isolated from tail tips from a minimum of 10 affected and 10 unaffected mice was pooled and subjected to a genome-wide scan using 48–80 simple sequence length polymorphic markers distributed throughout the genome. Samples used in the DNA pools were tested individually to confirm the map location [23].

### 2.4. Preparation of RNA Samples and Subsequent Analysis

Total RNA was isolated from whole eyes and brains of affected mutants and B6 mice using TRIzol Reagent (Life Technologies) per manufacturer's protocol. Total RNA was treated with RNase-free DNaseI (Ambion) and quantity was determined using a NanoDrop spectrophotometer (Thermo Scientific). RNA quality was evaluated with an Agilent Technologies 2100 Bioanalyzer. cDNA was generated using the Retroscript kit (Ambion).

Primers to sequence the coding region of the candidate genes were designed from exon sequences obtained from the Ensembl Database. RT-PCR was done using eye cDNA in a 24 *μ*L PCR reaction containing 1xPCR buffer (10 mM Tris-HCl pH 8.3, 50 mM KCl), 250 *μ*M of each dATP, dCTP, dGTP, dTTP, 0.2 *μ*M of each forward and reverse primer, 1.5 mM MgCl2, and 0.6 U Taq polymerase. The following PCR program was used: 94°C for 1 minute 30 sec followed by 35 cycles of 94°C for 30 sec, 55°C for 45 sec, and 72°C for 45 sec, and a final extension of 72°C for 2 minutes. PCR products were electrophoresed on a 1% agarose gel and visualized by ethidium bromide staining. DNA fragments were sequenced on an Applied Biosystems 3730XL (using a 50 cm array and POP7 polymer).

### 2.5. Histological Analysis

Mice were asphyxiated by carbon dioxide inhalation, and enucleated eyes were fixed overnight in cold methanol/acetic acid solution (3 : 1, v/v). The paraffin-embedded eyes were cut into 6 *μ*m sections, stained by hematoxylin and eosin (H and E), and examined by light microscopy.

## 3. Results and Discussion

### 3.1. Status of the EMR Program

Since its inception in the 1980s, the EMR program has identified and/or imported more than 100 mouse models with ocular abnormalities for research.  Table 1 lists some of the retinal degeneration mouse models of human disease developed and/or currently maintained in the EMR that are available to the Research Community. Other models are described on the EMR web page (http://eyemutant.jax.org/).

### 3.2. Status of the TVRM Program

The TVRM program was built upon the success of the Neuromutagenesis Facility (NMF) at The Jackson Laboratory, and 15 of the 60 mutant lines (Tables 2 and 3) in which a disease phenotype has been subsequently fixed as a coisogenic inbred strain by the TVRM program were first identified in screens conducted by the NMF. The remaining 45 TVRM lines were established by screening ~14,000 G3 mice for anterior and posterior segment abnormalities by indirect ophthalmoscopy and/or slit lamp biomicroscopy. Six of the 60 mutations (10%) are inherited in a dominant or codominant manner, and the remaining are recessive mutations. Forty six of the mutants have retinal phenotypes ranging between pan-retinal spots or patches, pigmentation defects, and/or attenuation of blood vessels with or without morphological changes that were detectable by light microscopy. Six of the mutant lines have reduced or absent ERG responses for either rod and/or cone cells without photoreceptor loss. Five mutant lines presented with vitreal fibroplasia and three with cataracts. Forty six of the mutations (23 reported in Table 3) have been localized to a chromosome, and the molecular basis has been identified for 23 of them (Table 2). Fourteen lines are still in the process of being mapped (data not shown). Nineteen of the 23 mutations in Table 2 were novel alleles in genes in which mutations had previously been reported. Some of these mutants are described below. It should be noted that the current bias for reoccurrences of mutations, herein referred to as remutations, versus identification of novel genes in Table 2 is probably due to the fact that once a mutation is mapped, candidate genes previously associated with an eye disease can be quickly sequenced. Regions containing no obvious candidate genes need to be narrowed further and/or all genes within the region may need to be sequenced to identify the disease-causing mutation.

Interestingly, new phenotypes were observed in 8 of the remutations that have been examined (see; [51–55], personal communication PMN). For example, outer segments (OSs) were either formed abnormally or did not initiate in retinas from homozygous *R* *p* *g* *r* *i* *p*1^*nmf*247^ mice [51]. This was in contrast to the *R* *p* *g* *r* *i* *p*1^t*mlTili*^ targeted null mutant, hereafter, *R* *p* *g* *r* *i* *p*1^−/−^ in which OS discs were formed and stacked vertically rather than horizontally [56]. Targeted alleles of *Lama1* were reported to be embryonic lethal [57, 58]. The ENU-induced allele, *L* *a* *m* *a*1^*nmf*223^, provides a viable, hypomorphic allele in which abnormalities in the adult animal could be examined. Clinically, vitreal fibroplasia and abnormal retinal vasculature were observed. Histologically, persistent hyaloid vessels and fibrous tissue were found in the vitreal space, and the inner limiting membrane was disrupted [52]. In an allelic series of mutations within the rhodopsin gene, light-induced retinal degeneration was observed. Heterozygous *R* *h* *o* ^*Tvrm*1^ and *R* *h* *o* ^*Tvrm*4^ mice raised in standard vivarium lighting did not exhibit any morphological changes until exposed to bright light [54]. Previously *Rho* alleles showed spontaneous and pan-retinal degeneration, even when mice were reared from birth in darkness [59].

### 3.3. New Alleles of *C* *r* *x* ^*tvrm*65^, *R* *p*1^*tvrm*64^, and *R* *p* *e*65^*tvrm*148^

#### 3.3.1. *C* *r* *x* ^*tvrm*65^

*tvrm65* segregates as a recessive mutation that is characterized by a pan-retinal, grainy fundus appearance that eventually progresses with age to patches of depigmentation within the central retina (data not shown). The mutation was mapped to chromosome (Chr.) 7 between flanking markers *D7Mit75* and *D7Mit190*. A single nucleotide polymorphic (SNP) marker (SNP ID: RS13479126) served to narrow the interval. *Crx*, a reasonable biological candidate gene, contained within the minimal interval, was examined for a mutation. 

**Figure 2:**
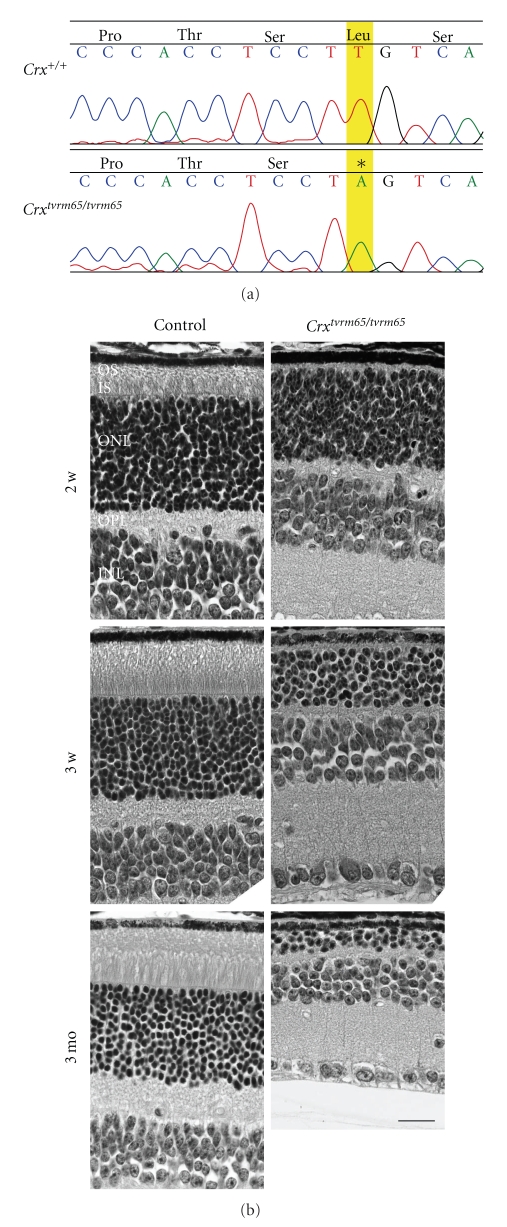
The mouse model *C* *r* *x* ^*tvrm*65^. (a) The mutation in homozygous *C* *r* *x* ^*tvrm*65^ causes a premature termination at aa residue Leu277. The mutated nucleotide is highlighted (b). Histology of control and *C* *r* *x* ^*tvrm*65^ mutant retina at P14, P21, and 3 months of age. OSs were absent at all ages in homozygous *C* *r* *x* ^*tvrm*65^, and progressive thinning of IS, ONL, and OPL was observed. OSs: outer segments, ISs: inner segments, ONL: outer nuclear layer, OPL: outer plexiform layer, INL: inner nuclear layer. Scale bar = 20 *μ*m.

CRX is an evolutionary conserved protein. Mice and humans share a 97% sequence similarity. To date, two *Crx *transcripts have been reported. The long isoform (Genbank nm_001113330) has 25 additional amino acids (aa) in its N terminus when compared to the shorter isoform (Genbank nm_007770). A T>A nonsense mutation identified in *C* *r* *x* ^*tvrm*65^ is located in the last exon and is expected to affect both isoforms. The *tvrm65* mutation is predicted to cause an early termination at Leu277 (TTG) of the 323 aa from the longer isoform or at Leu253 of a 299 aa product from the shorter isoform (Figure 2(a)). 

Phenotypically, *C* *r* *x* ^*tvrm*65^ mutants resemble the null mouse model in which the single homeodomain containing region [60] of *Crx* was targeted. Homozygous *C* *r* *x* ^*tmlClc*^ mice do not develop OS and photoreceptors degenerate. *C* *r* *x* ^*tvrm*65^ mutants show a rapid photoreceptor degeneration (Figure 2(b)). At postnatal day (P) 14 and P21, OSs were absent and inner segments (ISs) were rarely observed (Figure 2(b)). By P21, photoreceptor cell bodies were reduced to ~60% of controls. The outer plexiform layer (OPL) was also thinner, approximately 40% of controls. By 3 months of age, the OSs and ISs were absent and only 2~3 layers of outer nuclear layer (ONL) were remained. The photoreceptor degeneration observed in the *C* *r* *x* ^*tvrm*65^ mutants occurs more rapid than reported for the null allele [60]. This may, in part, be due to the difference in genetic background of the two alleles as *C* *r* *x* ^*tvrm*65^ was generated on a B6 background, whereas the previous null allele was described on a segregating B6 and 129Sb genetic background.

#### 3.3.2. *R* *p*1^*tvrm*64^

**Figure 3:**
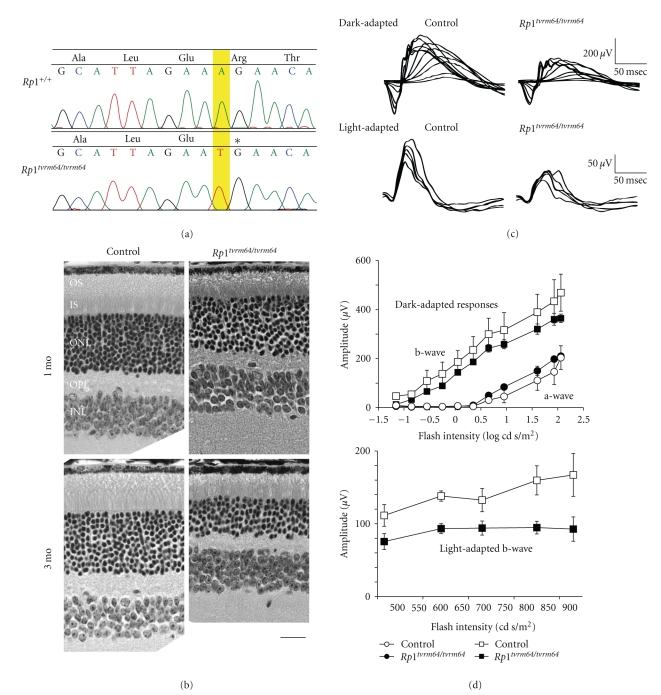
The mouse model *R* *p*1^*tvrm*64^. (a) Direct sequencing of control and *R* *p*1^*tvrm*64^ homozygous mutant identified an A to T mutation, predicting early termination at Arg522. The position of the mutation is highlighted and an asterisk indicates the termination. (b) The retinal morphology of control and *R* *p*1^*tvrm*64^ mice was examined at 1 and 3 months of age (mo). OSs: outer segments, ISs: inner segments, ONL: outer nuclear layer, OPL: outer plexiform layer, INL: inner nuclear layer. Scale bar = 20 *μ*m. (c) Electroretinogram of dark-adapted (scotopic) and light-adapted (photopic) control at 9 weeks of age and *R* *p*1^*tvrm*64^ at 4 weeks of age. (d) The amplitude of dark-adapted a and b-wave and light-adapted b-wave (±SEM, *n* = 3) of 4 weeks old *R* *p*1^*tvrm*64^ mice and age matched controls.

*tvrm64* segregates as a recessive mutation that is characterized by a grainy fundus appearance and attenuated retinal vessels (data not shown). The mutation mapped to Chr.1 between the centromere and *D1Mit427*, an interval in which *Rp1* resides. *Rp1* encodes a large protein of 2095 aa in mouse and 2156 aa in humans. RP1 localizes in the connecting cilia and appears to play a structural and/or functional role in molecular transport through the connecting cilia [61, 62]. Mouse RP1 shares 72% similarity with human RP1. Structurally, it has two ubiquitin homolog (UBQ) domains in its amino terminus. *Rp1* was tested for a mutation, as the phenotype of homozygous *Tvrm64* mutants was similar to that of mice carrying either of two targeted *Rp1* alleles, involving homologous recombination in which exons 2 and 3 were targeted (*R* *p*1^*tm*1*Jn*2^) [61] or a truncation after codon 662, *R* *p*1^*tm*1*Eap*^, analogous to the R667ter mutation in humans [62]. Direct sequencing of homozygous *R* *p*1^*tvrm*64^ retinal cDNA revealed an A>T transversion at nucleotide 1769 (Genbank nm_011283), creating a nonsense mutation in which Arg522 (AGA) is changed to a termination codon (TGA; Figure 3(a)). The mutation is localized adjacent to the two UBQ domains in RP1. 

The OS length of *R* *p*1^*tvrm*64^ mutant retina was approximately 50% shorter than WT controls at 1 month of age (Figure 3(b)). The difference in IS length between mutant and controls, however, was barely discernable at 1 month of age but was obviously shorter in *R* *p*1^*tvrm*64^ mutants at 3 months of age. The photoreceptor degeneration was progressive with little difference in cell body number in the ONL at 1 month of age but by 3 months, cell nuclei were reduced to ~50% in mutants in comparison to controls. In contrast, the photoreceptor morphology of *R* *p*1^*tm*1*Jn*2^ mice [61] appeared normal by light microscopy at P30 with comparable length of OS in mutant and controls. Also, *R* *p*1^*tm*1*Eap*^ mice [62] at P30 showed shorter OS lengths and a 1–2-layer reduction in ONL. Therefore, the disease progression in *R* *p*1^*tvrm*64^ at similar age appears to be more severe than observed in *R* *p*1^*tm*1*Jn*2^ mice but less severe than *R* *p*1^*tm*1*Eap*^ mice.

This difference between the models was also discernable functionally. At 1 month of age, dark-adapted ERGs of *R* *p*1^*tvrm*64^ mice were comparable to WT (Figures 3(c) and 3(d)). In *R* *p*1^*tm*1*Eap*^, these responses were significantly reduced at 4~5 weeks of age [62].

#### 3.3.3. *R* *p* *e*65^*tvrm*148^

**Figure 4:**
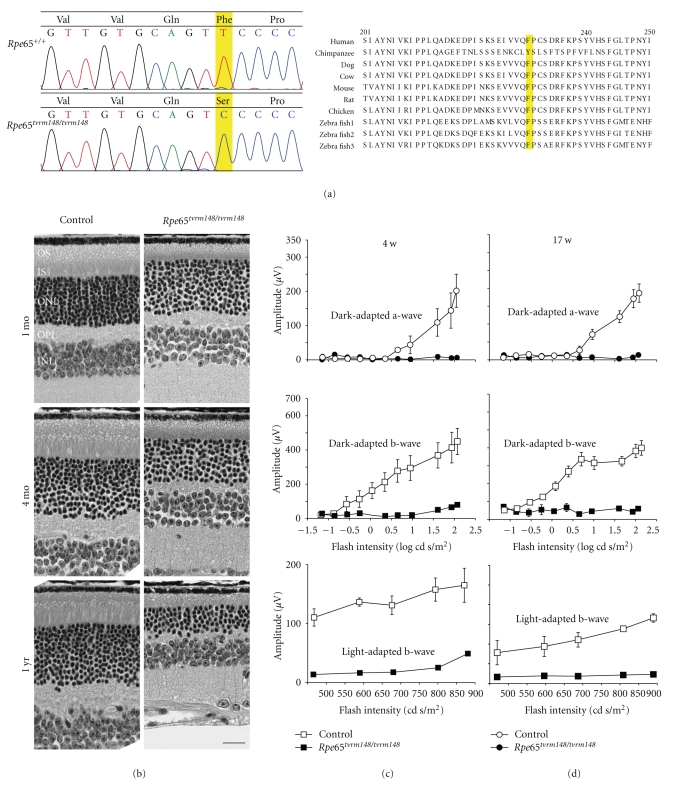
The *R* *p* *e*65^*tvrm*148^ mouse model. (a) Mutation analysis by direct sequencing revealed that the homozygous *R* *p* *e*65^*tvrm*148^ mouse harbored a missense mutation at aa residue 229, causing an amino acid change from Phe to Ser. The highlighted nucleotide indicates the mutation in the *R* *p* *e*65^*tvrm*148^ mouse (left). RPE65 protein is an evolutionarily conserved protein, and F229 is a nearly invariant residue from human to zebra fish (right). (b) Retinal morphology at 1 and 4 months and 1 year of age was analyzed by light microscopy. ONL thinning was progressive, and IS/OS was shorter than controls at all ages examined. OSs: outer segments, ISs: inner segments, ONL: outer nuclear layer, OPL: outer plexiform layer, INL: inner nuclear layer. Scale bar = 20 *μ*m. (c, d) Physiological retinal function was analyzed by ERG at 4 weeks (c) and 17 weeks of age (d). The plotted amplitude was obtained at 9 weeks from control and *R* *p* *e*65^*tvrm*148^ mice (c) or at 17 weeks from control and from homozygous *R* *p* *e*65^*tvrm*148^ mice. *N* = 3.

The recessive *tvrm148* mutation is characterized by late onset retinal spotting and by patches of depigmentation that is readily discernable by indirect ophthalmoscopy at 5 months of age (data not shown). The mutation mapped to Chr. 3 between markers, *D3Mit147* and *D3Mit19*. *Rpe65* was screened by direct sequencing for a mutation as it fell within the minimal interval identified, and the disease phenotype was similar to that reported for the *R* *p* *e*65^*tmlTmr*^ targeted knockout animal (herein referred to as *R* *p* *e*65^−/−^) [63] and *R* *p* *e*65^*rd*12^ [64] alleles. A T>C point mutation was found by direct sequencing of retinal cDNA from *R* *p* *e*65^*tvrm*148^ mice and is expected to generate a mutant protein with an F229S point mutation (Figure 4(a)). F229 is evolutionarily conserved from humans to zebra fish but interestingly not in chimpanzee (Figure 4(a)). 

The *R* *p* *e*65^*tmlTmr*^ mutant [63] had a nonfunctional rod ERG response due to the lack of 11-*cis*-retinal production in the RPE and showed disorganized rod outer segments. Another targeted allele mimicking a human R91W mutation was found in Leber Congenital Amaurosis (*LCA2*) patients (*R* *p* *e*65^*tmlLreb*^) [65], and a spontaneous model *R* *p* *e*65^*rd*12^ [64] showed a similar disease progression to that observed in *R* *p* *e*65^*tvrm*148^ mutants. Photoreceptors degenerated progressively in homozygous *R* *p* *e*65^*tvrm*148^ mouse from 1 month to 1 year of age, the latest time point examined (Figure 4(b)). At 1 month of age, OS and IS lengths were approximately 50% shorter than controls with no obvious thinning of the ONL. The photoreceptor nuclei were reduced in thickness by ~20% at 4 months and ~60% by 1 year of age. 

Like the three previously reported mouse models, *R* *p* *e*65^*tvrm*148^ exhibited severely impaired rod ERGs and relatively spared cone ERGs. Rod responses were absent by 4 weeks of age. However, cone b-wave ERGs were comparable to controls at 4 weeks of age but by 17 weeks, the amplitudes were reduced compared to controls (Figures 4(c)–4(d)).

## 4. Conclusions

### 4.1. The Utility of Spontaneous and Chemically Induced Mutations

Spontaneous or chemically induced mutations in mice provide a rich source of animal models. These mutations offer some advantages for the study of human genetic diseases and basic gene function over mutations obtained by homologous recombination. First, these mutations are generally identified because they cause a clinically relevant phenotype. By starting with a known phenotype, information about the physiological function of the mutant gene and its biomedical relevance is immediate. Second, the forward genetic approach has the potential for discovery of new genes involved in ocular development and function that were previously unappreciated. Further, spontaneous and chemically induced mutations may better model naturally occurring human genetic conditions. They produce a full and unbiased array of mutation types—single base pair changes or deletions, and in the case of spontaneous mutations, retroviral insertions, repeat sequence expansions, and chromosomal rearrangements. These mutations can create alternatively spliced transcripts or nonsense or missense reading frames. They can abolish all protein function (null), partially diminish function (hypomorphic), or change function (dominant negative or gain-of-function). Moreover, allelic series—collections of mutant alleles of the same gene—can provide domain specific information about protein function and information on alternatively spliced variants. Biomedically relevant phenotypes associated with some human genetic disorders may be revealed by the different alleles that are not replicated by knockout alleles. For example, whereas the null alleles of *Lama1* [57, 58] were embryonic lethal, the hypomorphic ENU *nmf223* allele allowed for the examination of ocular phenotypes in adult mice [52]. In another example, the *rd10* allele of *Pde6b* [66] identified by the EMR program has a later onset and slower rate of degeneration than the original *rd1* allele, thus allowing for the opportunity to test therapeutic strategies [67]. Finally, two phosphodiesterase 6a mutations first described by the TVRM program cause missense mutations that lead to different biochemical outcomes and rates of photoreceptor degeneration, suggesting a difference in the importance of the particular mutant residues to the function of the protein [55].

It should also be noted that spontaneous mutations occur on a wide variety of strain backgrounds, and chemical mutagenesis can be carried out in different genetic backgrounds. The observation of altered mutant phenotypes in different genetic backgrounds can provide a means for identifying interacting genes and molecular pathways of pathophysiology. For example, *N* *r*2*e*3^*rd*7^ was observable clinically only in the B6 genetic background [68], and a number of genetic backgrounds act to ameliorate the disease [69]. *C* *r* *b*1^*rd*8^ is observable clinically in the C3H/HeJ background but not in the B6 background [70], and the null mutation is phenotypically different on a segregating 129X1/SvJ and B6 background [71]. Finally, a wide variety of disease phenotypes are observed in *rd3* [27] and *G* *n* *b*1^*rd*4^ [28] in different strain backgrounds, indicating interactions with genetic background modifiers. The variation in genetic background enables discovery of modifiers and gene interactions and could be essential to the discovery of important mutant phenotypes and potential targets for therapeutic intervention.

### 4.2. The Future of the EMR and TVRM Programs

In the future, the EMR will continue to screen for spontaneous mutations in the large production colonies at The Jackson Laboratory. The mutants identified in the TVRM program will be incorporated into the EMR distribution colonies as the molecular bases of the mutations are identified. Finally, sensitized chemical mutagenesis screens are planned that will uncover pathways important in retinal development, maintenance, and function.

## Figures and Tables

**Table 1:** Mouse retinal mutants maintained in the Eye Mutant Resource (EMR) at The Jackson Laboratory.

Model	Mode	Gene	Chr.	Clinical Phenotype
*rd1*	AR	*Pde6b*	5	Early onset, severe retinal degeneration [24]
*pcd*	AR	*Agtpbp1*	13	Slower retinal degeneration associated with Purkinje cell degeneration [25]
*nr*	AR	*UN*	8	Progressive retinal degeneration with hyperactive ataxic behavior (nervous) [25]
*Rd2*	AD	*Prph2*	17	Slow progressive retinal degeneration [26]
*rd3*	AR	*Rd3*	1	Retinal degeneration, beginning at 3 weeks of age [27]
*Rd4*	AD	*Gnb1*	4	Autosomal dominant retinal degeneration [28]
*Tub*	AR	*Tub*	7	Retinal degeneration, hearing loss, and late-developing obesity, also known as *rd5 *[29]
*mnd*	AR	*Cln8*	8	Early onset retinal degeneration with a late-onset progressive motor neuron degeneration [30]
*rd6*	AR	*Mfrp*	9	Small, white retinal spots and progressive photoreceptor degeneration [31]
*rd7*	AR	*Nr2e3*	9	Retinal spots and progressive photoreceptor degeneration [32]
*rd8*	AR	*Crb1*	1	Focal photoreceptor degeneration [33]
*Rd9*	XD	*UN*	X	Progressive retinal white spotting and degeneration [33]
*rd10*	AR	*Pde6b*	5	Early onset, mild retinal degeneration [34]
*rd11*	AR	*Lpcat1*	13	Retinal degeneration with white retinal vessels at 4 weeks of age [35]
*rd12*	AR	*Rpe65*	3	Poor ERG response and late onset retinal degeneration [36]
*rd14*	AR	*UN*	18	Slow retinal degeneration with white retinal spots [37]
*rd15*	AR	*UN*	7	Retinal degeneration with retinal outer plexiform dystrophy [38]
*rd16*	AR	*Cep290*	10	Early onset retinal degeneration [39]
*rd17*	AR	*Gnat1*	9	Poor rod ERG response and slow retinal degeneration [40]
*cpfl1*	AR	*Pde6c*	19	Cone photoreceptor function loss-1 [41]
*Cpfl2*	AD	*UN*	3	Cone photoreceptor function loss-2 with white retinal spots [42]
*cpfl3*	AR	*Gnat2*	3	Cone photoreceptor function loss-3 [43]
*Cpfl4*	AD	*UN*	17	Cone photoreceptor function loss-4 [44]
*cpfl5*	AR	*Cnga3*	1	Cone photoreceptor function loss-5 [45]
*cpfl6*	AR	*Hcn1*	13	Cone photoreceptor function loss-6 [46]
*cpfl7*	AR	*UN*	19	Cone photoreceptor function loss-7 [47]
*nob2*	XR	*Cacna1f*	X	Anatomical and functional abnormalities (no b-wave-2) in the outer retina [48]
*nob3*	AR	*Grm6*	11	Retinal functional abnormalities (no b-wave 3) [49]
*arrd2*	AR	*Mdm1*	10	Age-related retinal degeneration-2 [50]

AR: autosomal recessive, AD: autosomal dominant, XR: X-linked recessive, UN: unknown.

**Table 2:** Mouse mutants from the Translational Vision Research Models (TVRMs) program in which the molecular basis for the disease phenotype has been identified.

Model	Mode	Gene	Chr.	Clinical Phenotype
*tvrm64*	AR	*Rp1*	1	Juvenile onset retinal degeneration
*nmf12*	AR	*Mertk*	2	Late onset slow degeneration
*tvrm148*	AR	*Rpe65*	3	Late onset retinal degeneration
*nmf192*	AR	*Nphp4*	4	Early rapid retinal degeneration
*nmf364*	AR	Pde6b*	5	Early rapid retinal degeneration
*nmf449*	AR	Pde6b*	5	Early rapid retinal degeneration
*Tvrm1*	AD	*Rho*	6	Light inducible retinal degeneration [51]
*Tvrm4*	AD	*Rho*	6	Light inducible retinal degeneration [51]
*Tvrm144*	AD	*Rho*	6	Light inducible retinal degeneration
*tvrm65*	AR	*Crx*	7	Early rapid retinal degeneration
*tvrm27*	AR	*Trpm1*	7	No B-wave
*tvrm89*	AR	*Myo6*	9	Attenuated ERG
*tvrm84*	AR	*Grm1*	10	Attenuated ERG
*nmf246*	AR	*Uchl3*	14	Juvenile onset retinal degeneration
*nmf247*	AR	*Rpgrip1*	14	Early rapid retinal degeneration [52]
*nmf5a*	AR	*Pfnd5*	15	Early rapid retinal degeneration
*nmf240*	AR	*Clcn2*	16	Early rapid retinal degeneration [53]
*nmf223*	AR	*Lama1*	17	Vitreal fibroplasia, vascular abnormalities [54]
*tvrm124*	AR	Tulp1*	17	Early rapid retinal degeneration
*nmf282*	AR	*Pde6a*	18	Early rapid retinal degeneration [55]
*nmf363*	AR	*Pde6a*	18	Early rapid retinal degeneration [55]
*tvrm58*	AR	Pde6a*	18	Early rapid retinal degeneration
*tvrm32*	AR	Hps1*	18	Pigmentation defect

*Established by complementation testing.

**Table 3:** Mouse mutants from the Translational Vision Research Models (TVRMs) program in which the molecular basis of the disease phenotype has not yet been identified.

Model	Mode	Chr.	Clinical Phenotype
*tvrm9*	AR	1	Retinal spots
*tvrm113*	AR	4	Retinal spots, grainy fundus appearance
*Tvrm6 *	AD	7	Retinal spots
*tvrm116*	AR	12	Retinal spots, late onset
*tvrm111*	AR	14	Retinal spots
*nmf289*	AR	16	Retinal spots
*tvrm5*	AR	18	Retinal spots in central retina
*tvrm10*	AR	19	Retinal spots, coloboma, and vascular defects
*tvrm77*	AR	6	Central patches
*tvrm119*	AR	18	Retinal patches
*tvrm127*	AR	18	Retinal patches
*tvrm102*	AR	6	Grainy retina
*tvrm101*	AR	10	Grainy retina
*nmf67*	AR	7	Fine web-like fundus appearance
*Tvrm122*	AD	3	Shiny flecks
*tvrm64a*	AR	12	None, identified through histology, lamination defect
*tvrm111b*	AR	8	Abnormal ERG
*tvrm87*	AR	4	Vitreal fibroplasia
*tvrm114*	AR	4	Vitreal fibroplasia, cataracts
*tvrm53*	AR	7	Vitreal fibroplasia
*tvrm85*	AR	18	Vitreal fibroplasia
*Tvrm49*	AD	15	Cataracts
*tvrm129*	AR	13	Cataracts
